# A Subset of Secreted Proteins in Ascites Can Predict Platinum-Free Interval in Ovarian Cancer

**DOI:** 10.3390/cancers14174291

**Published:** 2022-09-01

**Authors:** Molly J. Carroll, Katja Kaipio, Johanna Hynninen, Olli Carpen, Sampsa Hautaniemi, David Page, Pamela K. Kreeger

**Affiliations:** 1 Department of Biomedical Engineering, University of Wisconsin-Madison, Madison, WI 53706, USA; 2 Research Center for Cancer, Infections and Immunity, Institute of Biomedicine, University of Turku, FI-20014 Turku, Finland; 3 Department of Obstetrics and Gynecology, Turku University Hospital and University of Turku, FI-20014 Turku, Finland; 4 Research Program in Systems Oncology, Research Programs Unit, Faculty of Medicine, University of Helsinki, FI-00014 Helsinki, Finland; 5 Department of Pathology, Helsinki University Hospital, University of Helsinki, FI-00014 Helsinki, Finland; 6 Department of Biostatistics and Bioinformatics, Duke University, Durham, NC 27710, USA; 7 University of Wisconsin Carbone Cancer Center, University of Wisconsin School of Medicine and Public Health, Madison, WI 53705, USA; 8 Department of Obstetrics and Gynecology, University of Wisconsin School of Medicine and Public Health, Madison, WI 53705, USA; 9 Department of Cell and Regenerative Biology, University of Wisconsin School of Medicine and Public Health, Madison, WI 53705, USA

**Keywords:** ovarian cancer, ascites, Lasso, platinum-free interval, model stability, robustness

## Abstract

**Simple Summary:**

Identifying proteins that correlate with better or worse outcomes may help to identify new treatment approaches for advanced high-grade serous ovarian cancer. Here, we utilize a machine learning technique to correlate the levels of 58 secreted proteins in tumor ascites with the time to disease recurrence after chemotherapy, which is known clinically as the platinum-free interval. We identify several candidate proteins correlated to shorter or longer platinum-free intervals and describe model analysis methods that may be useful for other studies aiming to identify factors impacting patient outcomes. Future validation of these factors in a prospective cohort would confirm their clinical utility, whereas a study of the mechanisms that they impact may identify new therapies.

**Abstract:**

The time between the last cycle of chemotherapy and recurrence, the platinum-free interval (PFI), predicts overall survival in high-grade serous ovarian cancer (HGSOC). To identify secreted proteins associated with a shorter PFI, we utilized machine learning to predict the PFI from ascites composition. Ascites from stage III/IV HGSOC patients treated with neoadjuvant chemotherapy (NACT) or primary debulking surgery (PDS) were screened for secreted proteins and Lasso regression models were built to predict the PFI. Through regularization techniques, the number of analytes used in each model was reduced; to minimize overfitting, we utilized an analysis of model robustness. This resulted in models with 26 analytes and a root-mean-square error (RMSE) of 19 days for the NACT cohort and 16 analytes and an RMSE of 7 days for the PDS cohort. High concentrations of MMP-2 and EMMPRIN correlated with a shorter PFI in the NACT patients, whereas high concentrations of uPA Urokinase and MMP-3 correlated with a shorter PFI in PDS patients. Our results suggest that the analysis of ascites may be useful for outcome prediction and identified factors in the tumor microenvironment that may lead to worse outcomes. Our approach to tuning for model stability, rather than only model accuracy, may be applicable to other biomarker discovery tasks.

## 1. Introduction

High-grade serous ovarian cancer (HGSOC) is the leading cause of death among women with gynecological cancer, with four out of five patients diagnosed with advanced disease [1]. Most patients diagnosed with ovarian cancer are treated with a chemotherapy regimen of a platinum-based agent and a taxane (e.g., carboplatin/paclitaxel). Whereas the majority of patients show at least a partial response to these therapies (resulting in median overall survival of 4.3 years [2]), 25% will be platinum-refractory in the primary setting [3] and 20% will become platinum-resistant with a recurrence within six months of the conclusion of chemotherapy [4]. For patients with primary platinum resistance, only 15% of resistant tumors respond to subsequent therapies [5]. The time between the conclusion of chemotherapy and the first recurrence, or the platinum-free interval (PFI) [6], is the most important prognostic factor for overall survival (OS) [7]. Therefore, identifying factors that correlate with a worse PFI may provide new avenues for therapeutic development. 

One approach to identifying such factors is to utilize statistical analysis to correlate clinical data to patient outcomes. For example, patients with larger volumes of ascites (greater than 2 L) had a significantly shorter progression-free survival (PFS) [8], whereas bowel/mesentery involvement has been associated with platinum resistance [9]. However, clinical-level measurements such as these may be reached by multiple different biological mechanisms. Therefore, other studies have examined the relationship between gene and protein expression and the PFI/PFS [10,11,12]. To date, these studies have utilized biopsies from the primary tumor, which is removed from the patient prior to adjuvant chemotherapy, rather than samples from the metastatic sites that remain following debulking. It is important to remember that in addition to the tumor cells, the broader tumor microenvironment (TME) includes immune cells (e.g., T cells, monocytes, macrophages, NK cells), fibroblasts, mesothelial cells, and adipocytes. The relative levels of these cells may vary between the primary tumor (which is most frequently analyzed but removed during surgical debulking) and the metastatic tumors (which are more likely to persist after surgical debulking). Recent studies have demonstrated that these cells within the TME support tumor cell proliferation [13,14], metastasis [15,16], and chemoresistance [17]. One mechanism to survey the broader tumor burden and contribution of the TME may be to analyze the ascites, which is present in the majority of patients with Stage III/IV disease. Ascites refers to the pathological accumulation of fluid that results from leaks in the peritoneal lining. This fluid circulates throughout the peritoneal cavity and therefore contains secreted factors from a variety of cells, essentially reflecting the broader TME of the solid tumor. This fluid is removed during cytoreductive surgery or by paracentesis to provide relief to the patient and slow disease spread [18]. The fact that it is readily removed in combination with its potential to reflect the solid tumor suggests that ascites could serve as a liquid biopsy.

Ascites has been documented to have increased levels of numerous cytokines, proteases, and growth factors, including MIP-1β [16], MMP-9 [19], and HB-EGF [20]. Discovery-level proteomic screening of ascites in ovarian cancer has suggested potential biomarkers of disease [21,22]; however, most of these analyses are underpowered due to an order of magnitude difference in the number of proteins identified and the total number of patients screened. One recent study characterized approximately 700 proteins in the ascites of 70 patients, identifying 346 that correlated with time to relapse [23]. Ensuring that a sufficient number of samples can be obtained for fully powered analysis requires substantial trade-offs in the ability to cast a broader net. For example, a model identified IL-6 and TNFα as predictive of progression-free survival, but the analytes were limited to 13 well-known cytokines/growth factors [24]. The balance between the number of possible analytes and the challenges of collecting sufficient samples remains an important challenge in the field of biomarker discovery.

We propose that ascites provides an opportunity to probe the interactions across all cells present in the TME and identify potential factors that drive ovarian cancer progression. To this end, we measured the levels of cytokines and growth factors in ascites collected at the time of diagnosis for 39 patients who had a recorded recurrence after primary treatment, with primary treatment being either neoadjuvant chemotherapy (NACT) or primary debulking surgery (PDS) prior to chemotherapy. Utilizing a Lasso-regularized regression model to predict the PFI [25], we identified key analytes that drove PFI prediction to better understand signaling that may lead to ovarian cancer recurrence. We also assess whether tuning for model stability rather than accuracy provides a mechanism to deal with studies that have more analytes than samples. 

## 2. Materials and Methods

### 2.1. Patient and Sample Characteristics

**Figure 1:**
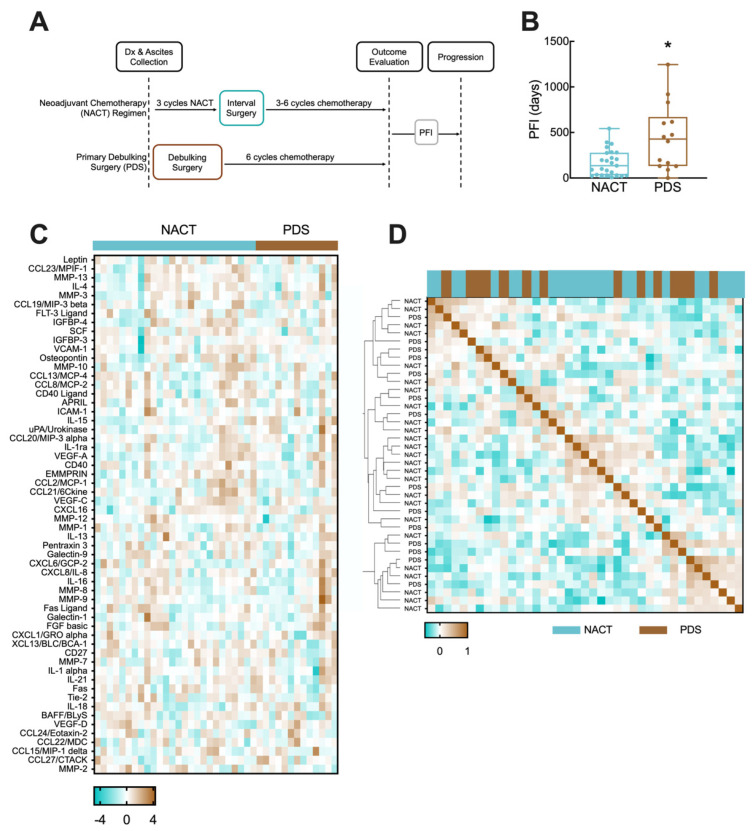
NACT and PDS regimens. (**A**) Schematic of NACT and PDS treatment regimens and measurement of PFI. (**B**) Box and whisker plot comparison of PFIs in the NACT and PDS subcohorts. * indicates *p* = 0.0123 by Mann–Whitney test. (**C**) Heatmap of Yeo–Johnson power transformation-normalized analyte concentrations for patients grouped by treatment regimen. (**D**) Similarity matrix for analyte concentrations based on Pearson correlation.

The patient cohort consists of patients treated for HGSOC at Turku University Hospital. All participating patients gave written informed consent. The study and use of clinical material were approved by The Ethics Committee of the Hospital District of Southwest Finland (ETMK), which consists of an unselected cohort of HGSOC patients with FIGO stage of at least IIIB collected between 2009 and 2017. Cell-free ascites samples were acquired from patients at the time of diagnosis. Ascites was recovered during a laparoscopic biopsy procedure performed on patients in the NACT cohort (*n* = 25) prior to the first cycle of chemotherapy. These patients then underwent up to four cycles of NACT, cytoreductive surgery, and an additional three to six cycles of chemotherapy. Patients in the PDS cohort (*n* = 14) underwent primary cytoreductive surgery, with ascites collected during this surgery, followed by at least six cycles of chemotherapy (see Figure 1A for an overview of this sampling procedure). Ascites was collected on ice, centrifuged, and the cell-free supernatant was frozen at −80 °C. Patient response to primary therapy and the time of disease progression was defined with RECIST1.1 and GCIG criteria [26,27]. All patients had documented dates of chemotherapy completion and tumor recurrence, as identified by radiographs. The PFI was defined as the interval of time between the completion of chemotherapy and disease recurrence. The sample collection is part of the HERCULES project (more information can be found at http://www.project-hercules.eu/ accessed on 7 June 2022). The HERCULES project prioritized the collection of NACT patient samples to examine the effects of chemotherapy; therefore, we had access to more NACT than PDS samples. 

### 2.2. Multiplexed Bead-Based Immunoassays

Multiplex bead-based immunoassays were purchased from R&D (Minneapolis, MN, USA) and included 116 analytes; panels were selected based on overlap with cancer-associated processes (e.g., immune cytokines, metalloproteinases) but were not necessarily known to be associated with HGSOC. Ascites samples were diluted according to manufacturer’s recommended dilutions for cell culture supernatant and analyzed on a Luminex 200 System (Luminex, Austin, TX, USA). Concentrations were determined from the standard curve by 5-point logistic fit. Some analytes were not detected uniformly across the patient set; analytes were removed from further analysis if detected in fewer than 75% of patients. This resulted in 57 measured analytes in the NACT group and 58 in the PDS group (see Supplemental Table S1 for the detected list; excluded analytes were generally excluded for both cohorts and samples were either above maximum or below minimum rather than a mix). Individual patient/analyte combinations that were below the detection limit of the assay were approximated as half of the detection limit multiplied by the assay dilution factor, whereas measurements above the maximum standard were approximated as two times the maximum multiplied by the assay dilution (see Supplemental Table S2 for details).

### 2.3. Data Preprocessing and Lasso Modeling 

Lasso (least absolute shrinkage and selection operator) is a machine learning tool that has been used in a variety of medical studies [28,29,30,31,32,33]. Like many machine learning tools, Lasso seeks a linear relationship between the independent variables (i.e., analyte levels in ascites) and a variable that is hypothesized to be dependent (i.e., PFI). To do so, Lasso seeks to identify an optimal subset of the independent variables by setting some analyte coefficients to zero [25]. If a zero coefficient improves the model score, the feature is dropped from the model. Through this process, the minimum set of features that are most strongly associated with the dependent variable is selected and the imbalance between a larger number of analytes relative to samples is partially addressed. This feature selection does not incorporate prior knowledge, allowing for the identification of unexpected relationships.

Within each cohort (NACT, PDS), analyte concentrations were log_10_-transformed. Analyte concentrations and patient PFI data were subjected to a Yeo–Johnson power transformation to transform the data to a normal distribution across the cohort [34]. This transformation does not use the label (patient outcome) and so does not introduce bias for machine learning. To further avoid bias, the standard evaluation methodology in machine learning when using a method with a hyperparameter (alpha in Lasso-penalized regression) and a small data set is nested leave-one-out cross-validation, defined precisely as follows. In the outer (evaluation) loop of cross-validation, a different example is held aside as a test case on each iteration. The model is trained on the remaining data, tested on the test example, and the error is recorded. At the end of the procedure, the errors are averaged. For root-mean-square error (RMSE), squared errors are summed, and averaged, and the square root is taken. For model training, the inner loop of cross-validation repeats the same procedure (with one fewer example total) for each possible setting of the hyperparameter, then chooses the setting that resulted in the lowest error, and trains a model with that particular setting on all the training data. To determine an optimal alpha penalty term for each cohort’s Lasso model, 201 alpha values ranging from 1 × 10^−5^ to 1 × 10^5^ were tested using scikit learn’s GirdSearchCV package (Python; Wilmington, DE, USA). The optimal alpha value was determined by the lowest RMSE using leave-one-out cross-validation (LOOCV), calculated using the following equation across *n* patients:

$$\mathrm{RMSE}=\sqrt{\frac{\sum_{1}^{n}{\left(\mathrm{Observed}\;\mathrm{PFI}-\mathrm{Pred}\;\mathrm{PFI}\right)}^{2}}{n}}\;$$

With the optimal alpha penalty term, a Lasso regression model (Python) was trained on the entire cohort and the model coefficients were analyzed further. To calculate the percent coefficient of variation *(*%CV) of model feature coefficients, the following equation was used taking into account mean and standard deviation (SD) values for a feature across all cross-validation folds:

$$\%\;\mathrm{CV}=\frac{\mathrm{mean}\;\mathrm{coefficient}}{\mathrm{coefficient}\;\mathrm{SD}}\times 100\%$$

## 3. Results

### 3.1. NACT and PDS Patients in Cohort Have Differential Time to Disease Recurrence

The cohort of ovarian cancer patients included 39 patients who received chemotherapy by NACT or following PDS and had a recorded recurrence after primary treatment (Figure 1A, Table 1). For both groups, the PFI was calculated as the interval between the end of the final cycle of chemotherapy and documented disease recurrence. Clinical data can be found in Table 1; all patients included in this study had a diagnosis of Stage III or IV HGSOC with ascites present at the time of diagnosis. Although it has been established that patients with larger volumes of ascites have a worse outcome [8], studies report that over 70% of Stage III/IV patients present with ascites [35,36], suggesting that ascites-based correlations would have broad applicability.

When using machine learning techniques such as Lasso regression, it is an advantage to have a large training cohort; however, in these types of models, it is assumed that samples and outcomes are independently and identically distributed. Whereas all patients were treated with carboplatin-based chemotherapy and ascites was collected prior to chemotherapy and surgery, NACT was selected for patients based on a clinical evaluation that suggested that the tumor was inoperable, likely due to greater tumor burden or location of metastases identified through pre-operative imaging. Therefore, we hypothesized that the PFI interval distribution for NACT and PDS patients would be different based on this selection bias. When we analyzed the distribution of the PFI between the NACT and PDS cohorts, their distributions were significantly different, with mean PFIs of 166 days and 447 days, respectively (Figure 1B, *p* = 0.0123).

This result suggested there may be differences in the two groups that would impact the ability to build a single model for both cohorts. We next measured the concentrations of cytokines, chemokines, and growth factors in the ascites of all patients (Figure 1C; Supplemental File S1). To determine if there were significant differences between the two cohorts, we first examined differences between NACT and PDS patients for individual secreted factors (Supplemental Table S2). This showed that only osteopontin was significantly different between the ascites of patients (elevated in NACT, *p* = 0.016, un-corrected two-tailed *t*-test), consistent with findings in prior studies [37]. However, within each treatment group, the coefficient of variation for each analyte was greater than 100% in 53 of the detected analytes (Supplemental Table S2). Therefore, we hypothesized that although there were only limited differences in population-level averages of each protein to potentially explain the difference in the PFI, there may be patterns of secreted proteins that differed between the two groups. Analysis of the protein expression using a similarity matrix demonstrated that PDS and NACT patients did not cluster separately, and there were multiple sub-clusters of NACT and PDS with similar expression patterns (Figure 1D). Therefore, we chose to build two separate models, one for NACT and one for PDS therapies, to predict the PFI from ascites analytes. Although the extent of debulking has been previously shown to predict patient outcome, we observed significantly longer outcomes for those with a <10 mm tumor remaining for the NACT group (*p* = 0.005), but not for the PDS group (*p* = 0.47). Given the inconsistency, we chose not to further divide our cohorts based on the remaining tumor burden.

### 3.2. Regression Model Predicts Disease Recurrence from Ascites Protein Levels for NACT Cohort

**Figure 2:**
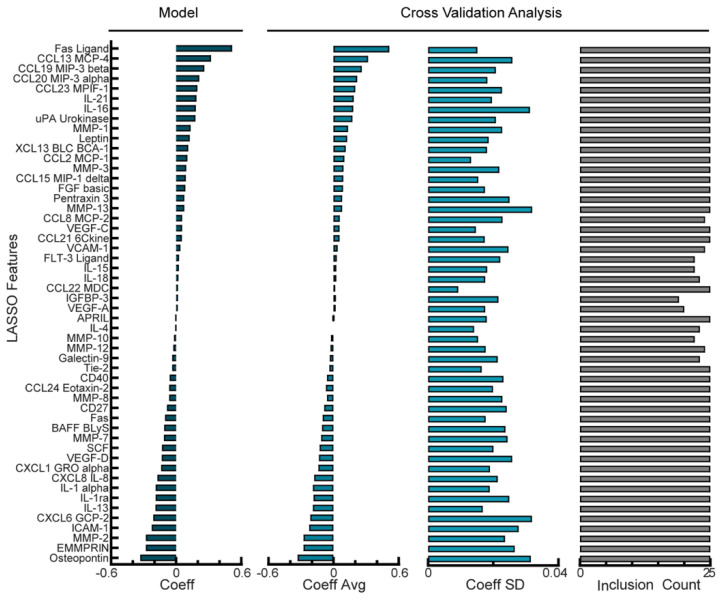
Regularized regression predicts PFI from analyte levels in ascites of NACT cohort. Coefficient for each analyte feature in the optimal Lasso model trained on all NACT patients. Cross-validation analysis includes average and standard deviation of coefficient values and the number of times each analyte was given a non-zero coefficient value across leave-one-out cross-validation folds in the optimal NACT model. Positive coefficients correspond to longer PFI.

The NACT cohort was comprised of 25 patients whose ascites had detectable, variable levels of 57 analytes screened for by multiplexed bead-based immunoassays. No single analyte showed a strong correlation (r^2^ > 0.5) with the PFI (data not shown). We next trained a Lasso model of the NACT cohort using tuned penalty parameters and analyzed model performance, features used in the model, and model stability. Using LOOCV with the tuned hyperparameters as described, the model had a root-mean-square error (RMSE) between the recorded and predicted PFI of 0.57 days (Supplemental Figure S1). By training the entire NACT cohort, the model used all 57 analytes that had been detected in at least one sample (Figure 2, left panel). Positively-weighted analytes, such as Fas ligand, CCL13, and CCL19 correlated with higher PFIs and negatively-weighted analytes, such as osteopontin, EMMPRIN, and MMP-2 were higher in patients with lower PFIs. When analyzing model stability using cross-validation, we found that the standard deviation for the features in the final model was relatively high compared with the average of the features during cross-validation (Figure 2, middle panels), with an average coefficient of variation of 135%. Additionally, only 46 of the 57 features used in the optimal model were used in all cross-validation folds (Figure 2, right panel). These model stability analyses suggest that whereas using the optimal penalty term produces a model with a very low error, the chosen features and their coefficients may be subjective to overfitting and lack robustness to predict future patient data.

### 3.3. Balancing Model Error Tolerance and Sparsity Increases Model Robustness for NACT Cohort

**Figure 3:**
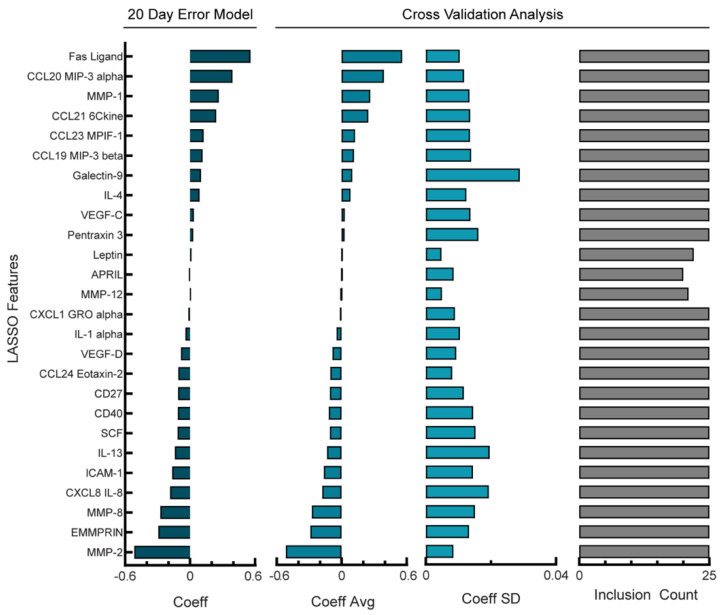
Reduction in features used in NACT model produces a more robust model. Lasso coefficient values and cross-validation error analysis for NACT model with RMSE = 18.6 days. Cross-validation analysis includes average coefficient values, standard deviation, and count of inclusion for the features retained in the full model across the 24 leave-one-out cross-validation folds. Positive coefficients correspond to longer PFI.

Our NACT model had high variability in which analytes were chosen during cross-validation as well as a large variance in coefficient values across the cross-validation folds. As an alternative, we next examined if models that allowed higher error would be more robust. Given that the error of our model had been less than one day whereas clinical timescales for patient outcome predictions are more typically on the order of weeks to months, we suspected that relaxing this tolerance could still lead to a useful model. We determined that a tolerable level of error would be 20 days, as only one patient had a PFI of less than 20 days. Next, we determined how increasing the penalty term in the Lasso model would impact the resulting model’s RMSE (Supplemental Figure S2A). By increasing the penalty term, we built a model with an RMSE of 18.6 days (Supplemental Figure S2B). Increasing the penalty term in Lasso should result in fewer non-zero coefficients being retained in the final model; as a result, this model used only 26 analytes as features (Figure 3, left panel). When we investigated the stability of this NACT model, the standard deviation of the features in relation to the average value during cross-validation was lower compared with the optimal model in Figure 2, with a percent coefficient of variation of 29% (Figure 3, middle panels). Finally, in the reduced analyte NCAT model, 23/26 of the analytes were used in all folds of cross-validation (Figure 3, right panel), an increased percentage compared with the optimal model (Figure 2, right panel). Therefore, although there is a higher PFI prediction error during cross-validation, we can be more confident in the analytes that were selected in the reduced analyte model as well as their coefficients. In this reduced analyte NACT model, Fas ligand and CCL20 were the highest-weighted analytes that correlated with the PFI, whereas an increased MMP-2 and EMMPRIN correlated with a shorter PFI (Figure 3, left panel). These analytes had appeared in the top three coefficients of the full model; the full model had osteopontin as one of the top three coefficients but osteopontin did not appear in the reduced model, possibly due to co-variation with another analyte. Finally, we did not observe changes in the sign for larger coefficients, suggesting that the model structures contain many similarities.

### 3.4. PFI Model of Analyte Levels in Ascites from PDS Cohort Depends Less Heavily on Sparsity to Produce Robust Predictions

**Figure 4:**
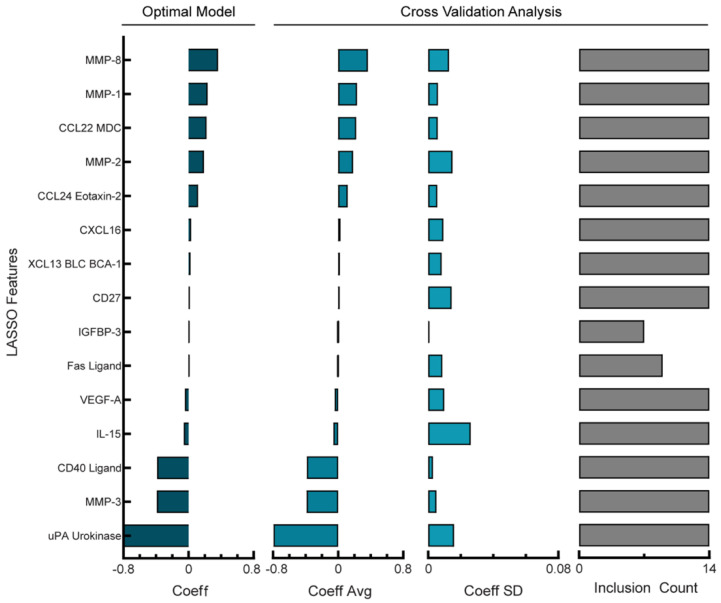
Regularized regression predicts PFI from analyte levels in ascites of PDS cohort. Lasso coefficient values and cross-validation error analysis for optimal PDS model (RMSE = 6.65 days). Cross-validation analysis includes average coefficient values, standard deviation, and count of inclusion for the features retained in the full model across the 14 leave-one-out cross-validation folds. Positive coefficients correspond to longer PFI.

The PDS cohort was comprised of 14 patients whose ascites had detectable levels of 58 analytes screened for using multiplexed bead-based immunoassays. Similar to the NACT group, no single analyte showed a strong correlation (r^2^ > 0.5) with the PFI (data not shown). Because the PDS cohort had fewer patients than the NACT cohort, we aimed to identify a model that balanced the PFI prediction error and overfitting. First, we determined that once again, 20 days would be a tolerable error as only one patient had a PFI less than 20 days. We compared how increasing the Lasso penalty impacted model prediction error and the number of features used in the model (Supplemental Figure S3A) and determined that although an optimal alpha parameter produced a model with RMSE of 6.65 days with 16 coefficients (Supplemental Figure S3B), increasing the penalty in the reduced analyte model led to an RMSE of 18.5 days using 15 coefficients (Supplemental Figure S3C). As the reduced analyte model only used one analyte less than the optimal model, we wanted to determine which of the models was more robust to cross-validation folds. First, we examined the model stability of the optimal PDS model using 16 features (Figure 4, left panel). When performing cross-validation analysis of the average and standard deviation of chosen feature weights (Figure 4, middle panels) we calculated an average percent coefficient of a variation of 41%; additionally, 13/16 features were used during all cross-validation folds (Figure 4, right panel). Next, we examined the stability of the reduced feature PDS model using 15 features (Supplemental Figure S3D, left panel). The ratio of the standard deviation and averages of the features during cross-validation (Figure S3D, middle panels) gave an average percent coefficient of a variation of 49%, and 13/15 features were used during all cross-validation folds (Figure S3D, right panel). As with the comparison of the NACT models, the reduced analyte model coefficients were of similar sign and magnitude, suggesting similar model structures. Therefore, although the reduced analyte PDS model used one fewer feature, it incurred a higher error and had less robust feature weights, and we concluded that the optimal parameter model provided the best fit and was more robust.

As noted, we built the NACT and PDS models separately based on observations of differences in the PFI and patterns of secreted proteins; however, it remained possible that the models would be of similar enough structure to cross-predict. However, the use of the 20-day error NACT model to predict the PFI for PDS patients resulted in an RMSE of 592 days. Likewise, the optimal PDS model was unable to predict the NACT PFI well, with an RMSE of 179 days. This suggests that the models may reveal information specific to PDS or NACT treatments.

## 4. Discussion

Our analysis of the cytokine, chemokine, and growth factor levels of this data set represents one of the largest quantitative analyses of ascites with associated clinical data. Characterization of samples collected prior to NACT is not well-represented in the literature. For example, a prior analysis of ascites using similar quantification methods profiled only 13 analytes compared with the 57–58 included here [24]. Some larger data sets have been generated for ascites from patients treated with PDS, including a proteomic analysis of nearly 700 analytes from 70 patients [23]. However, the signatures constructed with this data were only 77% accurate when boot-strapped, suggesting that the analysis was still underpowered. Here, we sought to find a middle ground and expanded our analyte number beyond a small multiplex assay of known regulators. Utilizing Lasso regression, we then developed and analyzed models correlating differences in ascites composition with the PFI for patients with stage III/IV HGSOC, identifying proteins that correlated with the PFI. Additionally, we implemented a procedure for model robustness analysis that may prove useful for other biomarker discovery tasks where the data set has similar limitations in patient numbers.

Several heavily weighted factors were related to tissue remodeling. For example, in the models built for ascites from the NACT cohort, MMP-2 was one of the most negatively weighted analytes (correlating to a short PFI). In a 3D organotypic model of ovarian cancer, it was found that MMP-2 plays a role in adhesion during the first steps of metastasis [38]. Additionally, a meta-analysis found that high levels of MMP-2 correlated with lower overall survival in ovarian cancer patients [39]. The overexpression of EMMPRIN (extracellular matrix metalloproteinase inducer/CD147) is associated with tumor angiogenesis in ovarian cancer [40] and has been correlated with worse outcomes for numerous cancer subtypes, including ovarian cancer [41]. EMMPRIN from mesothelial cells has been shown to induce gastric cancer cell invasion [42]. Consistent with this possible mechanism, EMMPRIN was slightly elevated when metastatic HGSOC tumors were compared with matched primary tumors [43]. Analysis of ascites would detect either tumor cell or mesothelial cell-derived factors. As both MMP-2 and EMMPRIN impact extracellular remodeling, this suggests that in vitro models for studying chemotherapy response in ovarian cancer may need to incorporate the stromal cells and extracellular matrix to better capture how tumor cells will respond. Although the PDS model had different specific analytes for the two most strongly correlated with a shorter PFI, we noted that it also had highly ranked proteases (MMP-3 and uPA urokinase), providing additional support that tumor remodeling during treatment may provide clues to recurrence. uPA urokinase levels have been shown to correlate with tumor stage and shorter overall survival in ovarian cancer [44].

Of course, the analysis of analytes correlated with a longer PFI also may reveal important biology to target in ovarian cancer treatment. For example, the Fas ligand was highly ranked in the NACT model. The Fas ligand/Fas receptor system has a complex role in the tumor microenvironment, with cytotoxic T cells expressing Fas ligand to induce apoptosis in tumor cells and tumor cells upregulating Fas ligand to attack infiltrating lymphocytes [45]. Fas ligand has also been shown to be upregulated following chemotherapy as a mechanism of resistance. Previous studies of ovarian cancer demonstrated that a high percentage of primary tumors in HGSOC are Fas ligand positive [46]. Additionally, soluble Fas ligand has been previously reported to be in vesicles in the ascites of ovarian cancer patients, although it is unclear if these were chemo-naïve samples or ascites collected following treatment [47]. Here, we only profiled ascites prior to chemotherapy, which clearly demonstrated that Fas ligand was present even before chemotherapy exposure. Interestingly, Fas ligand positively correlated in both the NACT and the PDS cohort, but the relationship was much stronger for the NACT patients. This could indicate that high levels of Fas ligand during early chemotherapy are beneficial. These high levels would be present in the NACT cohort during their treatment prior to debulking. As ascites is fully drained during debulking, the PDS patients would presumably have lower levels of Fas ligand for their initial chemotherapy treatments. Future validation in larger, prospective cohorts is needed to confirm the prognostic ability of these potential biomarkers.

A key advance of this study was our approach to address a common challenge in biomedical analysis—a data set that had a higher dimension in analytes relative to sample number [48]. This challenge is particularly common for ascites which must be processed fresh and not archived as part of standard clinical care. Although an obvious but non-trivial solution to this problem is to collect data from larger patient cohorts, we examined whether the tools from machine learning could be useful as well. For example, to combat the problem of overfitting, computational researchers have employed regularized methods to systematically reduce the number of features used to build the model. Here, we use an L1-regularized Lasso regression model to reduce the dimensionality of our dataset [25]; however, others have utilized other methods including regularized SVM [11] and Elastic Net [49] to build predictors for the response of tumors to therapies. With each regularized modeling technique comes the requirement for tuning hyperparameters. In our analysis, we utilized a leave-one-out cross-validation loop to determine an optimal penalty parameter for our Lasso regression model, a method often employed when there is insufficient data for tuning and training separately [50]. However, even with this approach, models can give an overly optimistic performance estimate when the number of training samples is small.

To address this limitation, we performed a more complete analysis of model stability (consistency of selected variables across cross-validation) than is typical in machine learning. By comparing the fraction of cross-validation folds in which an analyte was used and the standard deviation of the analyte’s weight across the folds, we can identify whether analytes are robustly being chosen for the model and whether their weight is consistent across folds of training data. For example, when we fit the NACT model with an optimal penalty parameter, we noted large standard deviations for the weights of each analyte as well as a large variability in which analytes were included in the model during cross-validation. However, this model’s error was less than a day, which is much more precise than clinical predictions will be expected to be. Therefore, we tested whether relaxing our error toleration would lead to a model that was more consistent. For the NACT model, this led to a model that used 31 fewer analytes, which is a potential advantage for translation due to the associated cost of measuring each analyte. Interestingly, for the patients treated in the PDS cohort, there were minimal differences in the number of analytes or robustness of the model. In Lasso, if predictive features are highly correlated, one is chosen arbitrarily. Our PDS patients had a large percentage of analytes that highly correlated with each other which may explain why the optimal model we explored had so many features eliminated from the model. Collectively, our approach of regularized regression and investigating model robustness should be considered during model development and the further refinement of predictive biomarkers.

## 5. Conclusions

Due to the challenges associated with treating ovarian cancer, much effort has been devoted to identifying new therapeutic approaches for the disease [51], including recent promising results for PARP inhibition for patients with BRCA1/2 mutations [10]. Ascites is well suited as a material to identify factors that may influence tumor response as it (1) contains analytes from the broader tumor microenvironment and (2) can be obtained from patients via biopsy/paracentesis or during debulking surgery [52]. Through the work presented here, we utilized Lasso regression to identify soluble proteins in the tumor microenvironment that could drive tumor recurrence. We also demonstrated an approach to tuning for model stability, rather than merely for model accuracy, which may be applicable to many other biomarker discovery tasks.

Finally, our results suggest that ascites profiling may be useful for outcome prediction for patients with high-grade serous ovarian cancer, which could lead to more informed treatment decisions. Our models were able to predict the PFI within a month, which is a range consistent with clinical forecasts. Such predictions could identify patients that are unlikely to respond to a standard of care, allowing the patient to prioritize the quality of life in their treatment plan or identify them as a candidate for experimental therapy in the frontline vs. recurrent setting. Further validation of the models identified here in independent, prospective cohorts would enable the use of ascites-based prognostication. In addition, a comparison of the levels of predictive analytes between ascites and serum may provide methods for more easily monitoring these factors during therapy.

## Figures and Tables

**Table 1:** Summary of clinical data for NACT and PDS cohorts.

	NACT	PDS	Statistical Comparison
N	25	14	
Age (years)	65.4 ± 7.0	66.1 ± 8.5	*p* = 0.73, 2-sided *t*-test, equal variance
Stage III	14	6	*p* = 0.51, Fisher’s exact test
Stage IV	11	8	
0–10 mm residual ^1^	16	9	*p* = 0.47, Fisher’s exact test
>10 mm residual	5	5	
<1 L ascites ^2^	5	4	*p* = 0.68, Fisher’s exact test
CA-125 (serum)	1900 ± 2900	2800 ± 4600	*p* = 0.53, 2-sided *t*-test, unequal variance; R^2^ = −0.16 vs. PFI
Total chemotherapy ^3^ cycles	6.1 ± 2.5	6.8 ± 1.4	*p* = 0.26, 2-sided *t*-test, equal variance
Carboplatin ^4^	4	1	*p* = 0.37, Fisher’s exact test
Bevacizumab maintenance	4	3	*p* > 0.99, Fisher’s exact test

^1^ residual information not available for all NACT patients. ^2^ Estimated at time of diagnosis, not available for 2 patients in each category. ^3^ For NACT, three cycles were completed prior to surgery; one patient had four cycles prior to surgery. ^4^ Most patients received carboplatin with paclitaxel—values here are for those who received carboplatin alone or carboplatin with doxorubicin.

## Data Availability

The data presented in this study are available in Supplemental File S1.

## Supplementary Materials

The following supporting information can be downloaded at: https://www.mdpi.com/article/10.3390/cancers14174291/s1, Figure S1: PFI prediction error in optimal NACT model; Figure S2: PFI prediction error in reduced analyte NACT model; Figure S3: Investigation of feature reduction in PDS model; Table S1: Analytes screened for in ascites; Table S2: Analyte characteristics for NACT and PDS cohorts. Raw data is available in Supplemental File S1.xlsx.
