# Supplementary material for: A Subset of Secreted Proteins in Ascites Can Predict Platinum-Free Interval in Ovarian Cancer
Source: Cancers (Basel). 2022 Sep 1;14(17):4291. doi: 10.3390/cancers14174291 (PMC9454800; doi:10.3390/cancers14174291)
Supplement: Supplementary file 1 [file cancers-14-04291-s001.zip › Supplemental figures and tables.pdf]

Supplementary Table S1. Analytes screened for in ascites

Analytes				
Consistently detected		Below limit		Above limit
APRIL	IGFBP-4	beta-NGF	IL-19	CD163
BAFF/BLyS	IL-1 alpha	CCL25/TECK	IL-2	CXCL10/IP-10
CCL13/MCP-4	IL-13	CCL26/Eotaxin-3	IL-22	IGFBP-1
CCL15/MIP-1 delta	IL-15	CCL3/MIP-1 alpha	IL-23	IGFBP-2
CCL19/MIP-3 beta	IL-16	CCL4/MIP-1 beta	IL-27	IGFBP6
CCL2/MCP-1	IL-18	CCL5/RANTES	IL-3	MIF
CCL20/MIP-3 alpha	IL-1ra	CCL7/MCP-3	IL-31	SCGF/CLEC11a
CCL21/6Ckine	IL-21	CX3CL1/Fractalkine	IL-33	Thrombospondin-2
CCL22/MDC	IL-4	CXCL11/ITAC-1	IL-34	TIMP-1
CCL23/MPIF-1	Leptin	CXCL2/GRO beta	IL-5	
CCL24/Eotaxin-2	MMP-1	EGF	IL-7	
CCL27/CTACK	MMP-10	G-CSF	LIF	
CCL8/MCP-2	MMP-12	GM-CSF	LIGHT	
CD27	MMP-13	HB-EGF	LT-alpha/TNF-b	
CD40	MMP-2	IFN-gamma	NRG1-beta 1	
CD40 Ligand	MMP-3	IL-1 beta	PDGF-BB	
CXCL1/GRO alpha	MMP-7	IL-11	Tau	
CXCL16	MMP-8	IL-12 p70	TGF-alpha	
CXCL6/GCP-2	MMP-9	IL-17A	TNF-alpha	
CXCL8/IL-8	Osteopontin	IL-17E/IL-25	TRAIL	
EMMPRIN	Pentraxin 3	IL-10		
Fas	SCF			
Fas Ligand	Tie-2			
FGF basic	uPA/Urokinase			
FLT-3 Ligand	VCAM-1			
Galectin-1	VEGF-A			
Galectin-9	VEGF-C			
ICAM-1	VEGF-D			
IGFBP-3	XCL13/BLC/BCA-1			

Supplementary Table S2. Analyte characteristics for NACT and PDS cohorts. For each analyte, the mean, minimum, maximum, standard deviation (SD), and coefficient of variation (%CV) are provided. The p-value for the uncorrected t-test comparing NACT and PDS groups indicated that only osteopontin was significantly different between the two groups.

	NACT Patient							PDS Patient						p-value
Analyte	Mean (pg/mL)	Min (pg/mL)	# Below Min	Max (pg/mL)	# Above Max	SD (pg/mL)	%CV	Mean (pg/mL)	Min (pg/mL)	# Below Min	Max (pg/mL)	# Above Max	%CV	(uncorrected)
APRIL	6868	2075		16635		3982	172	5581	2399		10518		200	0.250
BAFF/BLyS	4701	2658		6147		958	491	4306	2528		6598		289	0.397
CCL13/MCP-4	25	8		68		14	177	25	11		72		135	0.955
CCL15/MIP-1 delta	8086	5594		13751		2278	355	8534	6465		18353		258	0.666
CCL19/MIP-3 beta	415	75		1754		377	110	283	93		476		194	0.128
CCL2/MCP-1	900	150		4319		906	99	686	175		2668		102	0.415
CCL20/MIP-3 alpha	430	74		1706		456	94	767	15		3140		71	0.297
CCL21/6Ckine	754	226		2452		632	119	468	153		1215		134	0.077
CCL22/MDC	341	137		586		127	268	364	246		825		236	0.641
CCL23/MPIF-1	1511	902		2138		279	541	1450	1132		2069		460	0.559
CCL24/Eotaxin-2	633	147		1582		367	172	639	279		2425		108	0.974
CCL27/CTACK	313	160		611		89	350	362	194		610		277	0.240
CCL8/MCP-2	101	26		275		67	150	86	29		225		140	0.499
CD27	29566	15449		44384		8395	352	28895	17084		48940		281	0.841
CD40	1156	517		3143		518	223	1043	484		2559		193	0.540
CD40 Ligand	395	191		613		100	394	361	246		617		358	0.329
CXCL1/GRO alpha	685	117		3954		706	97	863	51		2182		121	0.471
CXCL16	3826	1461		4780	6	936	409	3912	2072		4780	5	400	0.795
CXCL6/GCP-2	1036	236		4751		947	109	1545	334		7004		90	0.333
CXCL8/IL-8	304	38		1158		246	124	438	37		2200	1	70	0.470
EMMPRIN	16827	4748		37180	1	8819	191	14131	5161		36486		164	0.369
Fas	25509	12680		39192		6750	378	22254	11371		35372		331	0.167
Fas Ligand	34	7		107		28	118	37	12		160		94	0.790
FGF basic	39	2	3	247		64	60	84	2	1	531		51	0.356
FLT-3 Ligand	283	169		543		98	290	253	155		389		359	0.266
Galectin-1	108831	44961		347918		54014	201	103863	49921		193775		274	0.741
Galectin-9	20210	7548		30200	4	6934	291	18631	8326		30200	2	260	0.518
ICAM-1	419259	122026		1824775		322980	130	446760	142614		1474031		128	0.814
IGFBP-3	417128	7371		862536		189494	220	445800	119674		791670		266	0.634
IGFBP-4	1018	190		3547		865	118	938	300		2544		129	0.761
IL-1 alpha	24	15		40		5	461	25	5	1	55		213	0.637
IL-13	886	598		1757		221	401	903	598		1211		474	0.803
IL-15	16	9		35		6	252	22	11		44		196	0.091
IL-16	679	282		1459		326	208	812	254		3937		82	0.644
IL-18	130	45		274		59	221	170	72		329		201	0.142
IL-1ra	4143	1088		15240	1	3407	122	4105	772		15240	1	94	0.978
IL-21	47	33	1	61		7	709	47	33	2	61		504	0.971
IL-4	74	52		85		8	952	73	50		101		603	0.835
Leptin	5443	501	1	16257		4513	121	5536	819		21596		100	0.959
MMP-1	1410	123		4490		1276	111	1115	119		5439		80	0.529
MMP-10	52424	2177		214780	3	73515	71	34460	2091		214780	1	58	0.416
MMP-12	1458	482		7359		1397	104	1149	30	1	2445		145	0.385
MMP-13	489	253		689		92	531	489	388		786		399	0.999
MMP-2	41342	32828		50669		4345	951	39851	34871		45379		1323	0.221
MMP-3	9102	3016		22770		4774	191	9559	2070		19503		174	0.801
MMP-7	34973	1832		172806		41252	85	38573	1832		145578		84	0.814
MMP-8	4683	752		18652		4321	108	12136	254		120380	1	37	0.430
MMP-9	679	135		1877		448	152	5137	286		52280		36	0.284
Osteopontin	80809	4656		353522		81260	99	37600	12690		95631		180	0.016
Pentraxin 3	31649	2205		100500		27909	113	21134	1588		55607		127	0.150
SCF	155	74		212		31	497	146	73		177		484	0.402
Tie-2	9992	4593		19772		3906	256	9061	5158		14711		314	0.407
uPA/Urokinase	2035	698		6042		1102	185	3644	792		19175		76	0.257
VCAM-1	1179047	12914		2345438		621961	190	940492	532497		2217587		197	0.196
VEGF-A	1173	206		4094		919	128	1339	102		4180	1	114	0.661
VEGF-C	683	258		1305		272	251	636	201		1052		275	0.585
VEGF-D	399	53		1910		399	100	546	103		2401		93	0.426
XCL13/BLC/BCA-1	909	237		2503		583	156	655	130		2541		101	0.247

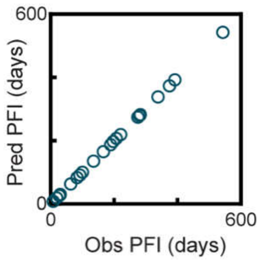

Supplemental Figure S1. PFI prediction error in optimal NACT model. Observed versus predicted PFI in days in NACT model using optimal penalty parameter (RMSE = 0.57 days).

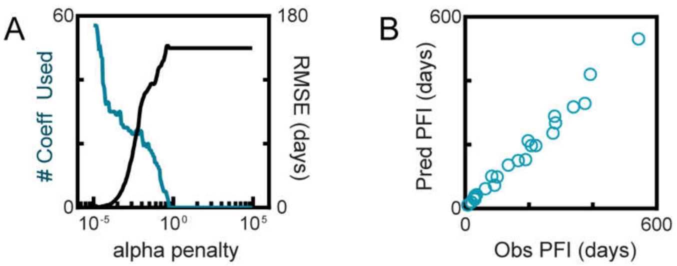

Supplemental Figure S2. PFI prediction error in reduced analyte NACT model. (**A**) Sweep of alpha penalty parameters demonstrating that an increased alpha penalty results in increased RMSE of PFI prediction and reduced number of features used by the model. (**B**) Observed versus predicted PFI in reduced feature NACT model using maximum penalty that resulted in RMSE less than 20 days.

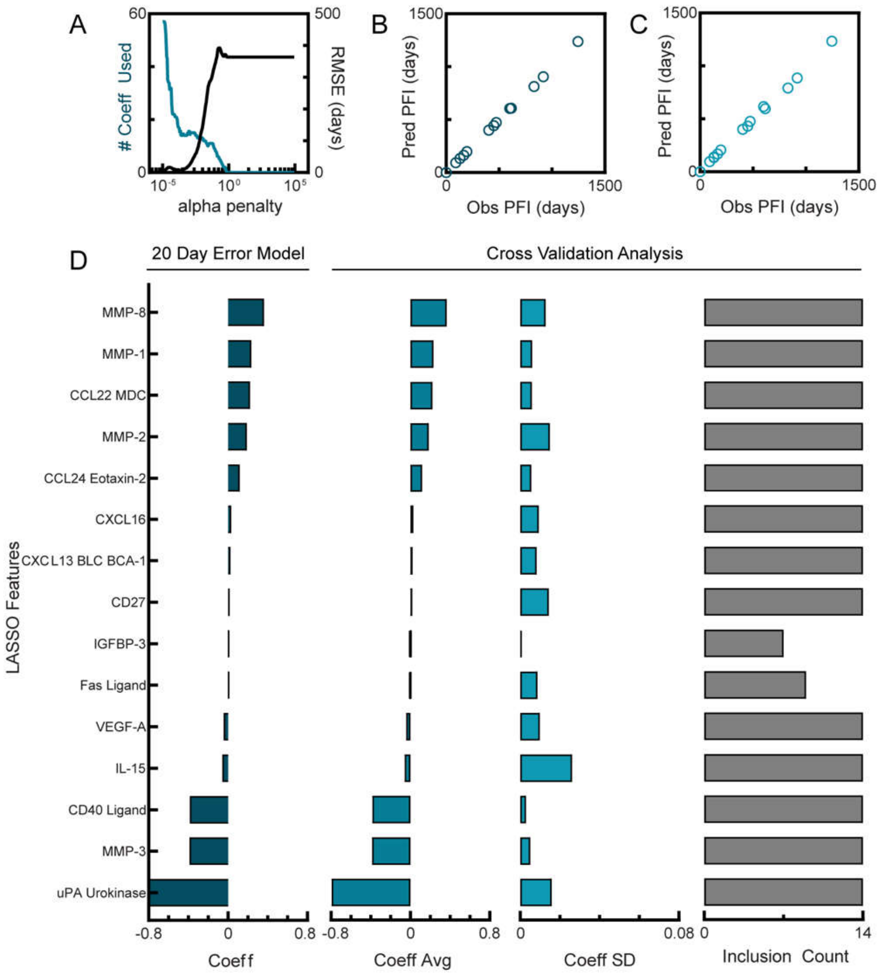

Supplemental Figure S3. Investigation of feature reduction in PDS model. (**A**) Sweep of alpha penalty parameters demonstrating that an increased alpha penalty results in increased RMSE of PFI prediction and reduced number of features used by the PDS model. (**B**) Observed versus predicted PFI in days in PDS model using optimal penalty parameter. (**C**) Observed versus predicted PFI in days in reduced feature PDS model using maximum penalty that results in targeted RMSE (18.5 days). (**D**) Lasso coefficient values and cross-validation error analysis for reduced feature PDS model. Cross-validation analysis includes average coefficient values, standard deviation, and count of inclusion for the features retained in the full model across the 14 leave-one-out cross-validation folds.
